# Immune Responses and Immunosuppressive Strategies for Adeno-Associated Virus-Based Gene Therapy for Treatment of Central Nervous System Disorders: Current Knowledge and Approaches

**DOI:** 10.1089/hum.2022.138

**Published:** 2022-12-14

**Authors:** Suyash Prasad, David P. Dimmock, Benjamin Greenberg, Jagdeep S. Walia, Chanchal Sadhu, Fatemeh Tavakkoli, Gerald S. Lipshutz

**Affiliations:** ^1^ Taysha Gene Therapies, Dallas, Texas, USA.; ^2^ Rady Children's Institute for Genomic Medicine, Rady Children's Hospital, San Diego, California, USA.; ^3^ Department of Neurology, O'Donnell Brain Institute, University of Texas Southwestern, Dallas, Texas, USA.; ^4^ Division of Medical Genetics, Department of Pediatrics, Queen's University, Kingston, Canada.; ^5^ Departments of Molecular and Medical Pharmacology and Surgery, Intellectual and Developmental Disabilities Research Center at UCLA, David Geffen School of Medicine at UCLA, Los Angeles, California, USA.

**Keywords:** adeno-associated virus, gene therapy, immunosuppression, CNS, innate immunity, adaptive immunity

## Abstract

Adeno-associated viruses (AAVs) are being increasingly used as gene therapy vectors in clinical studies especially targeting central nervous system (CNS) disorders. Correspondingly, host immune responses to the AAV capsid or the transgene-encoded protein have been observed in various clinical and preclinical studies. Such immune responses may adversely impact patients' health, prevent viral transduction, prevent repeated dosing strategies, eliminate transduced cells, and pose a significant barrier to the potential effectiveness of AAV gene therapy. Consequently, multiple immunomodulatory strategies have been used in attempts to limit immune-mediated responses to the vector, enable readministration of AAV gene therapy, prevent end-organ toxicity, and increase the duration of transgene-encoded protein expression. Herein we review the innate and adaptive immune responses that may occur during CNS-targeted AAV gene therapy as well as host- and treatment-specific factors that could impact the immune response. We also summarize the available preclinical and clinical data on immune responses specifically to CNS-targeted AAV gene therapy and discuss potential strategies for incorporating prophylactic immunosuppression regimens to circumvent adverse immune responses.

## INTRODUCTION

Gene therapy employing adeno-associated viruses (AAVs) is a promising approach to treat a variety of monogenic central nervous system (CNS) disorders. Clinical trials using AAV gene therapy have been completed or are ongoing for several CNS disorders including GM1 and GM2 gangliosidoses, Canavan disease,^1^ Batten disease,^2^ Sanfilippo syndrome,^3^ aromatic l-amino acid decarboxylase (AADC) deficiency,^4^ Parkinson's disease,^5^ spinal muscular atrophy (SMA),^6,7^ giant axonal neuropathy (GAN),^8^ Rett syndrome, and others.

AAVs are small (∼25 nm), nonenveloped viruses belonging to the *Parvoviridae* family.^9^ Twelve different naturally occurring AAV serotypes have been identified, with somewhat preferential tropism to different tissues depending on the target cell surface receptors and their corresponding binding sites present on the capsid.^9,10^ The single-stranded DNA genome of the AAVs encodes proteins required for replication (rep gene) and viral capsid components (cap gene) flanked by two inverted terminal repeats (ITRs).^11^ For AAV-mediated gene therapy, the rep and cap genes are replaced by the promoter, transgene product coding sequence, polyadenylation signal, and other regulatory elements of interest creating a transgene expression cassette.^10^

Multiple AAV serotypes including AAV1, AAV2, AAV5, AAV8, AAV9, and AAVrh.10 have been studied for the treatment of CNS disorders.^12^ AAVs are generally considered nonpathogenic, require helper viruses for replication, and in natural infections have relatively low rates of immune-mediated adverse events; however, some adverse immunological events have been observed in clinical trials with AAV gene therapy.^13–16^ Immune responses can be directed against the AAV capsid proteins, vector DNA (ITR, transgene, and regulatory elements),^17^ transgene product, or impurities in the vector preparation.^10^ Innate and adaptive immune responses can affect the safety of the patients and the durability of effective gene therapy.^18^

Considering the adverse immunological events observed in some of the previous trials of AAV gene therapy, it is becoming increasingly common to include an immunosuppression regimen, usually for a limited period of time. General immunosuppressants such as corticosteroids are most often used and have been combined with other drugs that specifically inhibit the function of B cells and/or T cells.^14,16,19–21^

Initial human clinical trials of CNS-targeted AAV gene therapy focused on intraparenchymal delivery, which used lower doses of the vector compared with other routes of CNS administration (reviewed in Hocquemiller et al; intraparenchymal dose range [total vector genomes] 9 × 10^10^–4 × 10^12^ vs. intrathecal [IT]/intravenous dose range 5 × 10^12^–3.3 × 10^14^).^12^ Few adverse immunological events have been reported with intraparenchymal delivery, presumably owing to the lower doses used and most of the vector remaining in the CNS.^12^ As direct delivery of the gene therapies in the brain parenchyma can often be challenging, other methods for delivery into the CNS are also being actively explored.

Some AAV serotypes can enter the brain across the blood–brain barrier (BBB) more easily than others, which raises the possibility of using systemic administration for CNS-targeted gene therapy.^22^ However, with this route of delivery, high vector doses resulting in widespread systemic exposure are required to achieve clinically relevant levels of transgene expression in the CNS, which may result in more pronounced immune responses^23^ and the potential for other end-organ injury such as hepatotoxicity.^24^ Delivery to the cerebrospinal fluid (CSF) through intracerebroventricular (ICV), IT, or intra-cisterna magna (ICM) administration reduces the systemic exposure and severity of immune-mediated adverse events; however, studies have demonstrated that these methods do not completely restrict the AAV distribution only to the CNS, as some outflow into the bloodstream occurs.^25–29^

Herein we review the innate and adaptive immune responses to the capsid, transgene and ITR DNA, and transgene product and how these responses can affect the safety and durability of AAV gene therapy. We also assess the reported adverse immunological events and the strategies currently being used to mitigate these events in AAV gene therapy clinical trials, with the objective of providing practical guidance and concepts that can be used when designing immunosuppression regimens to accompany CNS delivery of AAV gene therapy.

## INNATE IMMUNE RESPONSES TO AAV GENE THERAPY

The innate immune response is the first line of defense against pathogens or perceived pathogens (*e.g.,* AAV). Pathogen-associated molecular patterns (PAMPs) and molecules released from damaged host cells (damage-associated molecular patterns [DAMPs]) are recognized by pattern recognition receptors (PRRs) often expressed by innate immune cells (macrophages, monocytes, granulocytes, natural killer cells, innate lymphoid cells, and dendritic cells).^30,31^ One class of PRRs important for innate immunity against AAVs are Toll-like receptors (TLRs) present on or within cells. Activation of TLRs results in recruitment of adaptor proteins, such as myeloid differentiation protein 88 (MyD88), to the cytoplasmic portion of the TLR. This triggers a downstream signaling cascade (nuclear factor kappa B [NF-κB]) that leads to the production of proinflammatory cytokines (*e.g.,* type I interferons, interleukin [IL]-2, tumor necrosis factor α).^32^

Different TLRs have affinity for distinct classes of nucleic acids,^33^ and there are differences in exact nucleic acid specificity and TLR expression across species^34^ (*e.g.,* TLRs 11–13 are expressed in rodents but not in humans).^35^ TLR2 and TLR4 are expressed on the cell surface, where they detect viral lipoproteins and glycoproteins, whereas TLR3, TLR7, TLR8, and TLR9 are expressed in endosomal compartments and recognize nucleic acid variants normally associated with viruses. For example, TLR3 recognizes double-stranded RNA (dsRNA). It has been shown that AAV ITRs can have intrinsic promoter activity.^36,37^ When the plus-strand and minus-strand RNA generated from this intrinsic promoter activity anneal to form dsRNA in the cytoplasm of the AAV-transduced cells, the dsRNA can be recognized in immune cells by TLR3, which results in activation of the innate immune system or ubiquitously by viral RNA sensors (MDA5 and RIG-I) that may lead to programmed cell death.^17,38^

**Figure 1.**
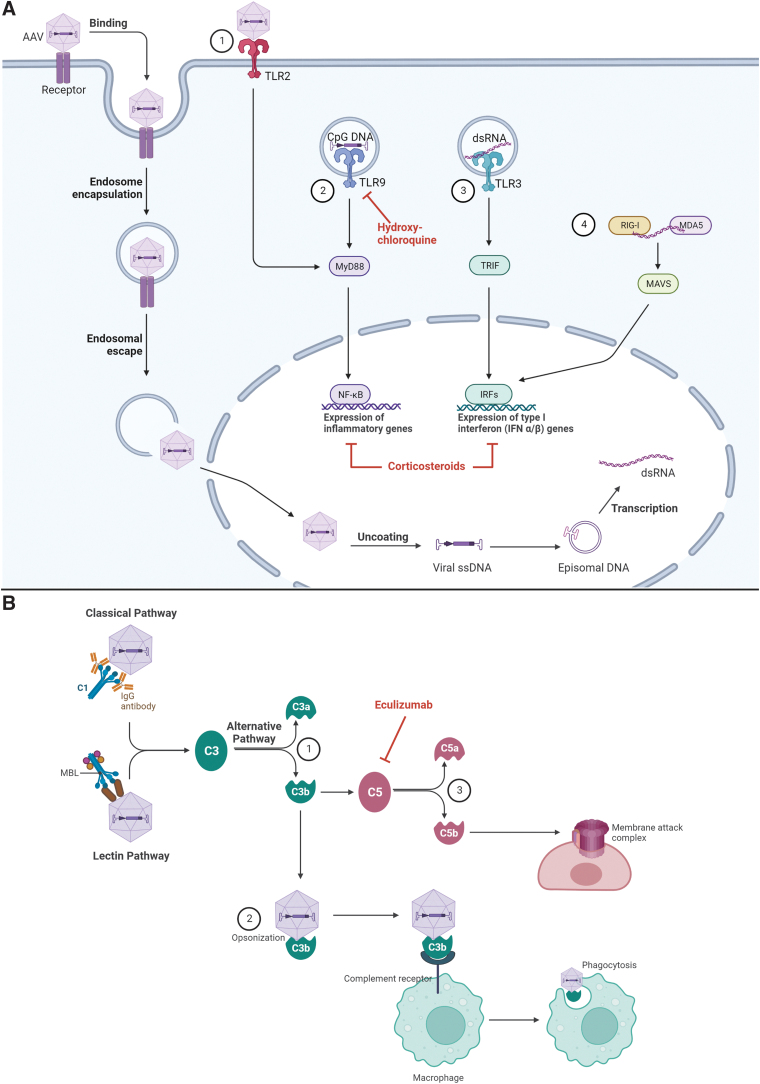
Overview of the innate immune response to AAV vectors. **(A)** At the cell surface, AAV capsids can bind TLR2 that recruits MyD88 and leads to activation of NF-κB and subsequent expression of genes that encode inflammatory cytokines ①. Within the cell, vector genomes can be exposed during endosomal trafficking and recognized by TLRs. CpG-rich AAV DNA activates intracellular TLR9 and the subsequent expression of genes that encode inflammatory cytokines via MyD88 and NF-κB ②, whereas dsRNA induces the expression of type I interferon genes via TLR3 ③ or RIG-1/MDA5 ④. Pharmacotherapies that can inhibit these pathways are shown in *red*, including corticosteroids and hydroxychloroquine. (**B)** Complement is activated via the classical, lectin, or alternative pathways. All pathways converge at the point of C3 activation and cleavage of C3 into C3a and C3b fragments ①. Opsonization of AAV by C3b fragments leads to activation of macrophages and phagocytosis of opsonized AAV ②. C3b also activates C5 and leads to the formation of the MAC in AAV-infected cells ③. Created with BioRender.com. Diagram is based on data available at the time of article development. AAV, adeno-associated virus; C1, complement component 1; C3, complement component 3; C5, complement component 5; CpG, cytosine-guanine dinucleotide; DNA, deoxyribonucleic acid; dsRNA, double-stranded ribonucleic acid; IgG, immunoglobulin G; MAC, membrane attack complex; MBL, mannose-binding lectin; MDA5, melanoma differentiation-associated protein 5; MyD88, myeloid differentiation primary response 88; NF-κB, nuclear factor kappa B; RIG-1, retinoic acid-inducible gene; TLR, Toll-like receptor.

TLR9 recognizes unmethylated cytosine-guanine dinucleotide (CpG) motifs (commonly observed in bacterial and viral DNA) within the vector DNA.^32^ Unmethylated CpG motifs in the AAV DNA are exposed during endosomal trafficking^31^ and on binding to TLR9 activate downstream signaling pathways (MyD88 to activate NF-κB and/or interferon regulatory factors) that lead to proinflammatory cytokine generation for immediate host defense (Fig. 1A).^31^ Proinflammatory cytokines facilitate immune cell recruitment and activation^39^ and stimulate CD8^+^ T cell responses.^40^ Zhu et al demonstrated that the TLR9-MyD88–induced production of type I interferon is essential for the activation of the CD8^+^ T cell response to the capsid and transgene-encoded product and is associated with the loss of transgene expression.^41^

Another arm of innate immunity is the complement system. Complement is activated through the classical, alternative, or lectin pathways, all of which lead to a common terminal pathway. In brief, the classical pathway is initiated when complement component C1 recognizes antigen-bound antibodies and undergoes conformational changes that generate a C3 convertase.^42,43^ The lectin pathway is activated on recognition of sugars on pathogen surfaces (*e.g.,* bacterial cell wall components). The alternative pathway begins when C3 that is spontaneously hydrolyzed encounters activated factor B and binds surfaces of pathogens, where it also acts as a C3 convertase.

Proteolytic activity of the C3 convertases produces C3a and C3b fragments. Soluble C3a fragments recruit macrophages and neutrophils to the site of infection, whereas deposition of C3b on AAV particles leads to enhanced phagocytosis, macrophage activation, immune complex clearance, adhesion of leukocytes to the vascular endothelium, proinflammatory cytokine production, and B cell activation. C3b can also form a C5 convertase, cleaving C5 to initiate the formation of the membrane attack complex (Fig. 1B).^43,44^

Considering recently reported adverse events, the U.S. Food and Drug Administration (FDA) Cellular, Tissue, and Gene Therapies Advisory Committee conducted a panel discussion on the safety of AAV-based gene therapy.^45^ Of particular importance was a recent clinical trial and postmarketing safety analysis for SMA, in which three patients experienced thrombotic microangiopathy (TMA) possibly owing to complement activation.^46,47^ All three patients were treated (one received a single dose of the complement inhibitor eculizumab) and eventually recovered.^47^ In addition, eculizumab was used to treat several patients in a Duchenne muscular dystrophy gene therapy trial who experienced acute kidney injury or thrombocytopenia resulting from complement activation despite taking daily glucocorticoids (NCT03362502).^48^ All the patients who experienced adverse events related to complement activation received a high dose of systemic AAV.

Although the mechanism of complement activation in these cases is unknown, Zaiss et al demonstrated that AAV-induced complement activation occurs only in the presence of immunoglobulin,^44^ raising the possibility that the classical pathway was activated on immune complex formation of C1 and the AAV capsid. They also showed that AAV capsids can interact with C3 fragments (opsonization), leading to macrophage activation and phagocytosis (Fig. 1B). Using C3 and complement receptor 1/2–deficient mice, this study concluded that the complement system is essential for the immune response to AAV.^44^ A recent *in vitro* study demonstrated a dose-dependent increase in levels of complement activation products C3a and C5b-9 in the presence of anti-AAV9 antibody and AAV9 capsid levels.^49^ Further studies are necessary to fully elucidate the mechanism of AAV-mediated complement activation, although the translatability of model systems, including nonhuman primates (NHPs), to the clinical setting is unknown owing to the differences in immune systems between species.

## ADAPTIVE IMMUNE RESPONSES TO AAV GENE THERAPY

**Figure 2.**
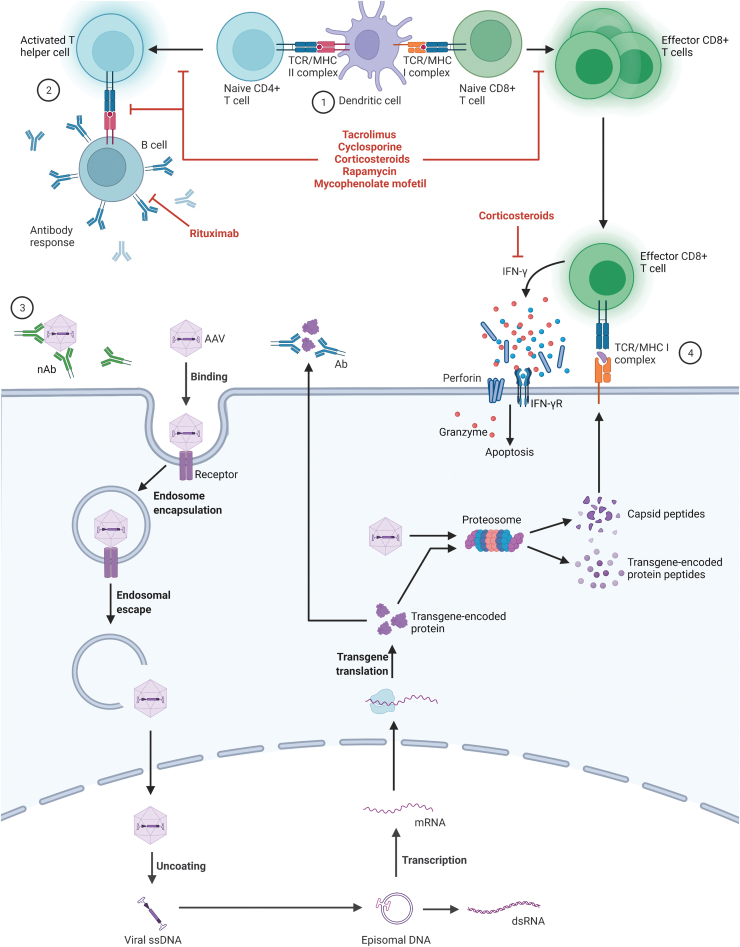
Overview of the adaptive immune responses AAV vectors. AAV capsids and transgene-encoded proteins within a transduced dendritic cell can be degraded by the proteosome and the resulting peptides are presented on MHCs leading to activation and proliferation of CD4^+^ and CD8^+^ T cells ①. Activated T helper cells signal B cells to produce antibodies directed at the capsid or transgene-encoded protein ②. nAbs against the AAV capsid inhibit interactions of AAV with its cellular receptor to prevent binding and transduction ③. Effector CD8^+^ T cells recognize and bind to other AAV-transduced cells presenting capsid or transgene-encoded peptides on MHC I molecules and initiate the cytotoxic T cell response ④. Pharmacotherapies that can interfere with these pathways are shown in *red* and include calcineurin inhibitors (tacrolimus and cyclosporine), corticosteroids, rapamycin, MMF, and rituximab. Created with BioRender.com. Diagram is based on data available at the time of article development. Ab, antibody; CD, cluster of differentiation; IFN-γ, interferon gamma; IFN-γR, interferon gamma receptor; MHC, major histocompatibility complex; MMF, mycophenolate mofetil; mRNA, messenger ribonucleic acid; nAb, neutralizing antibody; ssDNA, single-strand deoxyribonucleic acid; TCR, T cell receptor.

The innate immune response, as mentioned previously, acts as the first line of defense against the AAV capsid and leads to activation of the adaptive immune response. The adaptive response is highly specific to a particular antigen and takes longer to develop (several days).^50^ Humoral immunity is mediated by plasma cells secreting antigen-specific antibodies, including neutralizing antibodies (nAbs) that can block binding of AAVs to cell-surface receptors or interfere with the virus fusion mechanism to prevent endocytosis of the AAV. Antibodies to AAVs, including nAbs, often develop in humans resulting from exposure to naturally circulating AAVs.^51–54^ The antibodies are often cross-reactive among serotypes and the nAbs can block AAV cellular transduction, thus rendering gene therapy ineffective (Fig. 2).^52^

In addition to the preexisting anticapsid antibodies, including nAbs, the treatment itself can result in antibody development against both the capsid and the transgene-encoded protein. Most, if not all, patients without preexisting anticapsid antibodies are expected to seroconvert within days to weeks following systemic administration of AAV.^55^ Maturation of the B cell response leads to the production of lower affinity immunoglobulin (Ig)M followed by antigen-specific T cell–dependent isotype switching to higher affinity IgG antibodies.^55^ IgG antibodies may interact with cellular Fc receptors and potentially trigger death of AAV-infected cells or internalization and degradation of antibody-coated viral particles. They may also interact with complement-activating antibodies that could result in the lysis of AAV-infected cells as discussed previously (Fig. 1B).^56^

Cellular immunity directed against AAV gene therapy is mediated by CD4^+^ (helper) and CD8^+^ (cytotoxic) T cells. Antigen-presenting cells (APCs) take up AAV capsid antigens and/or transgene protein products and present them on class I major histocompatibility complex molecules (MHC I) or class II MHC (MHC II) molecules.^57^ APCs presenting antigens via MHC I activate CD8^+^ T cells, whereas APCs presenting antigens via MHC II activate CD4^+^ T cells.^57^ A potential intersection for the innate and adaptive immune systems occurs when plasmacytoid (pDCs) and conventional dendritic cells (cDCs) cooperate to cross-prime AAV capsid-specific CD8^+^ T cells. pDCs recognized the viral genome via TLR9, which leads to type I interferon production and subsequent activation of cDCs. Activated cDCs take up viral particles, present antigens via MHC I, and activate the CD8^+^ T cell response.^58^

The presence of capsid-specific CD8^+^ T cells (commonly measured by enzyme-linked immunospot [ELISpot] assay) were reported following administration of AAV encoding factor IX (FIX) in hemophilia B trials. The rise in CD8^+^ T cells was accompanied by a reduction in FIX levels over time.^16,59–62^ Studies have demonstrated that the loss of transgene expression in some tissues is owing to presentation of degraded capsid peptides or transgene product on MHC I, leading to the generation and activation of capsid-specific CD8^+^ T cells and subsequent destruction of the transduced host cell (Fig. 2).^57^ This cytotoxic T lymphocyte (CTL) response can also occur in humans previously exposed to AAV through natural infection owing to the expansion of memory CD8^+^ T cells that are reactivated by administration of AAV gene therapy.^60^

## HOST-SPECIFIC FACTORS THAT CAN DRIVE IMMUNOGENICITY IN AAV GENE THERAPY

Disease-specific underlying changes can affect the host response to treatment with AAV gene therapy. For example, the underlying genetic disorder or disease state may be accompanied by an already activated immune system, as is the case for neurodegenerative disorders associated with neuroinflammation, such as Alzheimer's disease.^63^ An activated immune system can impact the host immune response to the virus and can also alter the integrity of the BBB.^64^ Similarly, disease-specific changes in the target tissue can drive immunogenicity. For example, lysosomal storage disorders are characterized by the activation of microglia, neuroinflammation and, in some cases, leakage of the BBB.^65,66^ Disruption of the BBB allows peripheral immune cells to infiltrate the CNS and amplify or modify immune reactions.^67^ Diseases with ongoing inflammation may be more likely to have an increased immune response to AAV gene therapy.^67,68^

Immune responses to the transgene product can occur if a patient has not had previous exposure to the protein (during thymic selection and maturation), as is the case where the genetic defect results in no protein expression (referred to as cross-reactive immunological material [CRIM] negative) or the protein product does not contain key immunogenic epitopes.^69^ This suggests that the immunosuppressive regimen accompanying gene therapy should be tailored depending on whether a patient is CRIM negative or CRIM positive because the patient is more likely to experience an immunological consequence to the transgene when they are CRIM negative. This classification, however, is contingent on the reliability of protein expression predictions from various mutations and/or availability of experimental evidence. It is possible that some patients predicted to have partial protein expression may still recognize portions of the transgene product as a foreign antigen.^70^

## TREATMENT-SPECIFIC FACTORS THAT CAN DRIVE IMMUNOGENICITY IN AAV GENE THERAPY

The choice of administration route for AAV gene therapy can have a significant impact on immunogenicity. Some sites are thought to be relatively immune-privileged spaces, such as the eye and the CNS owing to the blood–retina barrier and BBB, respectively^23,71^; however, immune cells can cross the BBB^23,71^ and enter the CNS especially when neuroinflammation is present (*e.g.,* neurodegenerative and lysosomal storage disorders).^63–68^ In contrast to systemically administered gene therapies, direct administration into the brain parenchyma has the advantage of bypassing the BBB. However, compared with the cells distal to the site of administration, the cells proximal to the administration site will be transduced by a larger number of virions, resulting in a higher level of transgene-encoded protein expression than other parts of the brain.^72^ The consequent supraphysiological expression, in at least one study, has been shown to be associated with neurotoxicity in NHPs.^73^ It also cannot be ruled out that traumatic injury resulting from direct administration into the brain tissue could be proinflammatory.

Several important factors need to be considered when designing the optimal AAV therapy to minimize adverse immune responses. As indicated earlier, CpG islands of the vector DNA can trigger an immune response via activation of TLR9. Faust et al demonstrated that CpG-depleted genomes could evade the TLR9-mediated adaptive immune response in mice and represent a strategy for reducing AAV-associated immunity.^74^ More recently, this technique was used to produce a CpG-free ITR that resulted in a therapeutic micro-dystrophin vector when tested in mice.^75^ The authors speculate that the vector is less immunogenic, but further studies are needed to confirm the potential immunological advantage.^75^ dsRNA, formed when the AAV ITRs have promoter activity, can activate TLR3.^76^ Engineering the vector to weaken or eliminate ITR promoter function may decrease dsRNA formation and mitigate the immune response triggered by TLR3 activation.^43^

In addition to the genetic material carried by the vector, the immune system can recognize the transgene product as foreign. Promoters can be designed to mimic endogenous expression levels of the transgene product, such that a weak promoter may produce adequate transgene expression to be efficacious while mitigating toxic or immunological effects.^77^ Tissue-specific promoters can be used to drive transgene expression in target cells or organs^78^ and to limit expression in undesired tissues that could result in an immune response. For example, using promoters that are not active in professional APCs (*e.g.,* dendritic cells, Kupffer cells)^79^ could mitigate the cytotoxic T cell response by limiting antigen presentation and activation of effector T cells. However, CD8^+^ T cell responses directed against the transgene-encoded product can still occur in the absence of viral transduction and protein expression in APCs, whereby transgene-derived epitopes acquired by APCs from other types of transduced cells can be cross-presented and prime the anti-transgene product CTL response.^80,81^

nAbs to the AAV capsid can prevent binding to target cells and potentially inhibit transduction, rendering gene therapy ineffective. Modification of the AAV capsid to eliminate nAbs epitopes is a novel strategy that can be used to increase transduction efficiency and reduce nAb-mediated immune responses.^82^ The formation of antigen–antibody aggregates can also trigger the classical complement pathway leading to a type III hypersensitivity reaction.^83^ Capsid design could also be used to alter AAV tropism, reducing the titer of virus required for efficient transduction and decreasing potential adverse effects caused by high-dose therapy.^82^

The manufacture and purity of AAV-based gene therapy products are critical for reducing immunogenicity. Potential process- and product-related impurities associated with vector preparation include empty capsids, residual proteins from host cells and helper viruses, and encapsulated host cell nucleic acids or helper virus DNA.^84–86^ In terms of reducing immunogenicity, residual proteins and nucleic acids derived from the cell culture system used to produce the AAV should be minimized with the use of good manufacturing principles and high-quality purification techniques. Improved analytical methods to ensure accurate detection and quantification of impurities in the final vector preparation are essential to prevent manufacturing low-purity material.^86,87^

AAV vectors can be produced in mammalian (*e.g.,* HEK293, HeLa) or insect (Sf9) cells and can differ in their impurity profiles and posttranslational modifications of the AAV capsid proteins.^88^ For example, the use of insect cells can result in packaged insect cell DNA within the AAV vector product and subsequent expression of insect cell polypeptides in transduced cells, increasing the risk of transduced cell immunogenicity.^84^ The removal of empty capsids in AAV preparations is also recommended. Empty capsids are devoid of the transgene and convey no therapeutic benefit but can still elicit innate and/or adaptive immune responses. Studies have demonstrated that the AAV capsid can trigger dose-dependent immune toxicities whereby more significant adverse events are associated with high systemic doses of AAV.^59,61^

## DELIVERY APPROACHES TO THE CNS

The majority of CNS-targeted AAV gene therapy clinical trials have used intraparenchymal administration directly into the brain tissue through burr holes in the skull and stereotaxic delivery.^89^ Delivery through this method was used in multiple trials in, for example, Canavan disease,^1^ Batten disease,^2^ AADC deficiency,^4^ mucopolysaccharidosis,^89^ Sanfilippo disease,^3^ Parkinson's disease,^90^ and Alzheimer's disease.^91^ This approach generally required lower doses of viral vector and resulted in reduced off-target distribution into peripheral tissues, which can reduce the potential immune response to gene therapy.^90,92^

In addition to stereotaxic delivery, several trials are using systemic intravenous administration of AAV owing to certain serotypes having the ability to cross the BBB, the most common of which is AAV9.^22^ Systemic administration may appear advantageous because it is noninvasive, has a lower risk of infection and complications associated with the procedure, and can be used in diseases involving lesions in multiple brain regions that require broad therapeutic gene expression unable to be achieved with intraparenchymal administration.^93^ However, trials for SMA have been conducted using this method and were accompanied by hepatotoxicity and transient thrombocytopenia.^7^ Thus, several barriers to systemic administration need to be overcome, including peripheral toxicity and the innate and adaptive immune responses.

**Figure 3.**
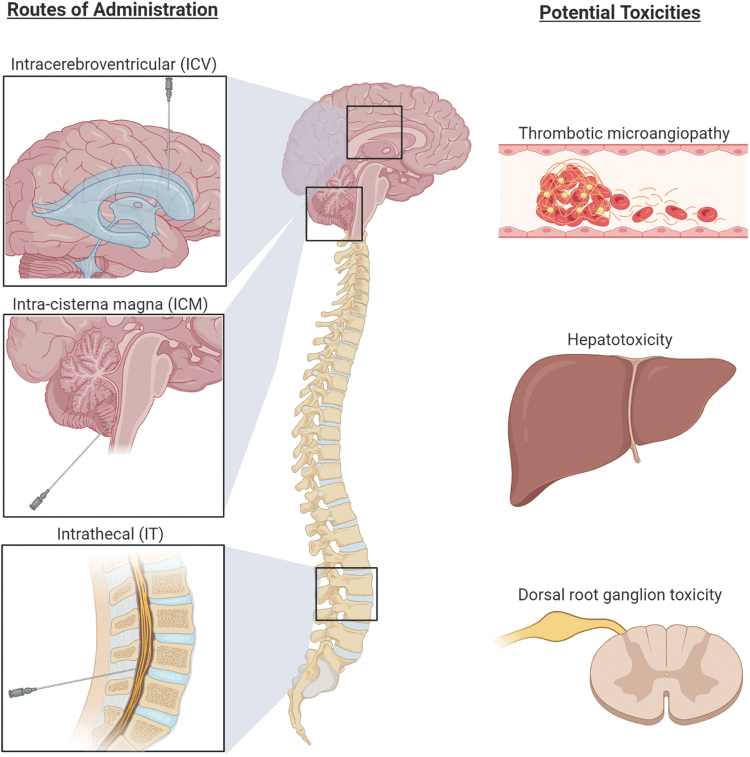
CNS routes of administration for AAV gene therapy and potential toxicities to monitor. Delivery of AAV gene therapy vectors to the CSF via ICV, ICM, or IT administration reduces the systemic exposure and severity of immune-mediated adverse events. Potential toxicities that should be monitored include TMA, hepatotoxicity, and dorsal root ganglion toxicity. Created with BioRender.com. CNS, central nervous system; CSF, cerebrospinal fluid; ICV, intracerebroventricular; ICM, intra-cisterna magna; IT, intrathecal; TMA, thrombotic microangiopathy.

CNS diseases that require targeting multiple regions or a wider AAV distribution in the brain may benefit from direct delivery into the CSF through the ventricles (ICV), cisterna magna (ICM), or spinal canal (IT), although biodistribution may vary depending on the specific route of administration (Fig. 3). Preclinical studies have established that most AAV serotypes enter the systemic circulation and transduce peripheral tissues following CSF administration.^25,26,29,94–96^ However, lower doses of AAV are needed to effectively transduce neuronal tissue through CSF administration, thus resulting in lower systemic exposure in comparison with IV delivery, potentially limiting systemic immune responses.^23^

Multiple clinical trials using IT delivery are currently underway (GAN [NCT02362438], infantile GM2 gangliosidosis [NCT04798235], Parkinson's disease [NCT03976349], Batten disease [NCT04737460, NCT02725580, NCT03770572, NCT04273243], SMA [NCT03381729, NCT05089656, NCT04042025], SMA associated with respiratory distress and Charcot–Marie–Tooth disease type 2S [NCT05152823], Tay-Sachs disease and Sandhoff disease [NCT04669535], and Rett syndrome).^97^ ICM delivery is being used in a trial for Parkinson's disease (NCT04127578)^98^ and in two trials for GM1 gangliosidosis (NCT04273269, NCT04713475).^99^

## IMMUNE RESPONSES TO CNS-TARGETED AAV GENE THERAPY

Immunological events have been reported in both preclinical studies and clinical trials for CNS-targeted AAV gene therapy. Most published AAV gene therapy studies for CNS diseases used intraparenchymal administration, so there are limited clinical data available regarding the immune responses that occur when administration to the CSF is used (ICV, IT, ICM). Intraparenchymal administration of AAV in trials has been well-tolerated overall. Although anticapsid antibody levels increased following AAV administration in several of these studies, they were not associated with any adverse events, clinical symptoms, or significant immunological events.^1,91,100–108^

Data regarding immune responses to IT administration of AAV gene therapy in humans are emerging from an ongoing trial of AAV9-GAN for the treatment of GAN.^8^ An early rise in anti-AAV9 nAbs and an elevation in CSF white blood cells (pleocytosis) that were not associated with any clinical or neuroimaging findings of neuroinflammation have been reported.^8^ These findings occurred in the presence of immunosuppression by prednisolone. Thereafter, subjects in the clinical trial also received rapamycin (and tacrolimus if CRIM negative) and appeared to have a reduced anticapsid T cell response, suggesting that T cell–mediated immunosuppression may reduce such antibody responses and CSF pleocytosis.^8^

Preclinical studies of CNS-administered gene therapy can provide some insights for the design of future clinical trials. A study in NHPs found that inflammation of the dorsal root ganglia can occur following IT administration of onasemnogene abeparvovec-xioi (Zolgensma),^46^ prompting the FDA to place a partial hold on a clinical trial testing IT administration in humans (this has since been lifted; NCT03381729).^109^ In clinical trials of onasemnogene abeparvovec-xioi for SMA, TMA occurred in three patients and was speculated to have possibly resulted from an innate immune response via activation of the alternative complement pathway.^47^

In a separate study, ICM administration of AAV9-hIDUA (for Hurler syndrome) in NHPs resulted in asymptomatic degeneration of some sensory neuron cell bodies in the dorsal root ganglion, marked CSF pleocytosis, hind limb weakness, degeneration of lumbar motor neurons, and infiltration of B and T lymphocytes into the dorsal root ganglia.^94^ Of importance, immune suppression using a combination of mycophenolate mofetil (MMF) and rapamycin did not eliminate these histopathological findings.^94^

Additional studies in NHPs indicate that neurotoxicity is a concern with CNS administration of AAVs. AAV9-green fluorescent protein administered via ICM was associated with moderate lymphocyte pleocytosis that correlated with higher CNS transduction.^95^ Bilateral infusion in the thalamus combined with ICV administration of AAVrh8-cmHexα/β (for Tay-Sachs/Sandhoff disease) in an NHP model resulted in dyskinesias, ataxia, loss of dexterity, and histopathology showing severe white and gray matter necrosis along the injection track. Of interest, antibodies against the transgene-encoded protein did not develop in the NHPs in this study; however, high levels of the transgene products (both α and β hexosaminidases) and their increased enzyme activities suggest that neurotoxicity may have been owing to transgene overexpression.^73^

Inflammation in the CNS can be initiated by microglia or by mononuclear cell infiltration. Activated peripheral T cells can traffic into the CNS in response to peripheral antigens, and B cell–mediated humoral responses can be initiated in the periphery or within the CNS.^23^ This and the studies listed previously support that, beyond close clinical observation for changes in neurological function, the following factors should be monitored in CNS-targeted AAV gene therapy trials: (1) antibodies against the capsid and transgene in blood; (2) T cell response against the capsid and/or transgene; (3) pleocytosis in the CSF; (4) markers of inflammation in the CSF; (5) assessment of BBB leakage; (6) neuroimaging to evaluate inflammation-related changes; (7) liver transaminases and bilirubin to evaluate hepatotoxicity; and (8) coagulation (with a focus on platelet counts and perhaps platelet function) to monitor for TMA. In addition, to complement the clinical neurological testing, nerve conduction studies with a focus on sensory nerve testing are important to perform at baseline and sequentially throughout the study to look for changes related to dorsal root ganglia function (Fig. 3).

Owing to the prevalence of hepatotoxicity observed in clinical trials weeks to months following gene transfer,^46,77,110^ it is advised that liver enzymes and function are measured by laboratory testing and the patient undergoes a clinical examination. Elevations in liver enzymes have been successfully reduced with corticosteroids in many cases^110,111^; still, hepatoxicity is associated with potential serious risk to the patient, underscoring the need for careful monitoring and prompt treatment. Recent reports of TMA observed in postmarket safety data of onasemnogene abeparvovec-xioi^47^ and in Duchenne muscular dystrophy clinical studies^48^ suggest that platelet counts should be monitored for thrombocytopenia, as early recognition and treatment are crucial for patient well-being and outcome.^47^

## APPROACHES TO IMMUNOSUPPRESSION

Initial trials of AAV gene therapy used a reactive approach to the administration of corticosteroids for instances of elevated liver enzymes suggestive of liver injury that were, in some cases, believed to be associated with AAV capsid-specific cytotoxic T cell response.^59^ Corticosteroid treatment typically resolved the elevation of liver transaminases.^46,59^ Owing to these findings, subsequent clinical trials incorporated prophylactic immunosuppression regimens that included one or a combination of pharmacotherapies (Table 1). Corticosteroids (prednisone, prednisolone, and methylprednisolone) bind to glucocorticoid receptors and modify transcriptional signaling that results in global anti-inflammatory and immunosuppressive effects.^112^ Corticosteroids exert these effects through multiple mechanisms including downregulation of TLR expression, suppression of proinflammatory cytokines, and upregulation of anti-inflammatory cytokines.^113^

**Table 1.** Immunosuppressive agents used in adeno-associated virus gene therapy studies

Immunosuppressant	Molecular Target	Mode of Action	Adverse Effects
Corticosteroids	Glucocorticoid receptor	Reduction of proinflammatory cytokines and chemokines^112^	Osteoporosis, metabolic disease, increased risk of cardiovascular disease^112,121^
Rapamycin (sirolimus)	mTOR	Suppression of cytotoxic T cell and helper T cell activation, Treg generation, suppression of B cell and T cell proliferation and differentiation^114,115^	Thrombocytopenia, dyslipidemia, mucositis, impaired wound healing, proteinuria^122,123^
Mycophenolate mofetil	Type II inosine monophosphate dehydrogenase	Suppression of B and T cell proliferation^116^	Gastrointestinal toxicity, leukopenia, infection^124^
Tacrolimus	Calcineurin/IL-2	Inhibition of T cell activation and proliferation and inhibition of T helper cell-dependent B cell response^117,125^	Abnormal renal function, hypertension, diabetes mellitus, fever, CMV infection, tremor, hyperglycemia, leukopenia, infection, anemia, bronchitis, pericardial effusion, urinary tract infection, constipation, diarrhea, headache, abdominal pain, insomnia, paresthesia, peripheral edema, nausea, hyperkalemia, hypomagnesemia, and hyperlipemia^126^
Rituximab	CD20	Induction of CD20^+^ B cell apoptosis^118,127^	Infusion-related reactions, skin and mouth reactions, hepatitis B virus reactivation, progressive multifocal leukoencephalopathy, febrile neutropenia, pyrexia, pneumonia, anemia, infection, tumor lysis syndrome^127,128^
Eculizumab	C5	Inhibition of complement activation^129^	Fever, high blood pressure, blood clots, anemia^130^
Hydroxychloroquine	TLR9	Inhibition of TLR9-mediated responses to viral DNA^119^ Inhibition of lysosomal activity that can prevent MHC-mediated antigen presentation^131^	Gastrointestinal effects, retinopathy, cardiomyopathy, cardiac conduction effects^131,132^

AAV, adeno-associated virus; C5, complement component 5; CD20, cluster of differentiation 20; CMV, cytomegalovirus; IL, interleukin; MHC, major histocompatibility complex; mTOR, mammalian target of rapamycin; TLR, Toll-like receptor; Treg, regulatory T cell.

Other immunosuppressants used in AAV gene therapies include rapamycin (also known as sirolimus), MMF, calcineurin inhibitors (cyclosporine, tacrolimus), and rituximab. Rapamycin inhibits the cell-cycle kinase mammalian target of rapamycin to suppress cytotoxic T cell proliferation, T helper cell differentiation, and at higher doses, B cell proliferation and differentiation.^114,115^ Antimetabolites such as azathioprine and MMF inhibit inosine monophosphate dehydrogenase, the rate-limiting enzyme for guanosine nucleotide synthesis that is upregulated in activated lymphocytes, thereby suppressing T and B cell proliferation.^116^

Cyclosporine and tacrolimus inhibit the signaling phosphatase calcineurin leading to suppression of IL-2 transcription, which is necessary for T cell proliferation, regulatory T cell maturation, as well as expansion and cytotoxic effects of effector T cells.^117^ The monoclonal antibody rituximab limits antibody production by targeting CD20 on B cells to induce apoptosis.^118^ Another pharmacotherapy being explored in preclinical trials is hydroxychloroquine, which inhibits TLR9 ligand binding and downstream signaling to prevent TLR-mediated T cell activation and proinflammatory cytokine production.^119^

It is important to consider the safety profile of immunosuppressants to ensure that the mitigation strategy does not result in additional adverse events. Dose, schedule, and length of treatment also impact the overall safety profile. Table 1 includes the most common adverse events associated with each immunosuppressant. In addition, immunosuppressed patients are more susceptible to bacterial, fungal, and viral infections; so, careful monitoring and a strategy for prophylaxis or managing infectious events while receiving immunosuppressive therapy is essential.

## CLINICAL AND PRECLINICAL STUDIES USING PROPHYLACTIC IMMUNOSUPPRESSION

A recent systematic review revealed that corticosteroid use was only reported in 46 of 149 AAV gene therapy clinical trials examined.^120^ Those studies that did report corticosteroid use can be classified into prophylactic (incorporated in all patients by default), reactive (incorporated at the investigator's discretion), or therapeutic (to resolve certain adverse events) administration.^120^ In our review of the data incorporated herein (Table 2), we focused on trials that used one or a combination of immunosuppressive therapies that were administered before or at the time of AAV dosing and continued post-AAV dosing. Although the number of published clinical trials of AAV gene therapy using corticosteroids and other immunosuppressants is limited, this approach is rapidly evolving, and we anticipate that the number of trials incorporating immunosuppressive therapies will continue to grow.

**Table 2.** Immunosuppressive regimens used in adeno-associated virus gene therapy studies

Disease/Target	Species	Route of Administration	Immunosuppressants Used	Immunosuppression Regimen	Immunologic Outcome	Adverse Events	Reference
Mucopolysaccharidosis type IIIB syndrome	Human (*n* = 4)	Intraparenchymal	Tacrolimus (0.2 mg/kg) MMF (1,200 mg/m^2^)	Immunosuppression started 14 days before AAV therapy. MMF maintained for 6 weeks. Tacrolimus tapered for 66 months	Persistent T cell response to the transgene detected over 66 months of follow-up, but no apparent impact on transgene expression	None reported	Gougeon (2021)^133^
Hemophilia A	Human (*n* = 15)	IV	Corticosteroid (40–60 mg/day)	Immunosuppression started 3 weeks before AAV therapy and gradually tapered. Corticosteroids were given reactively to decrease ALT	Capsid- and transgene-specific immune responses detected but not associated with adverse events or changes in efficacy	Transient elevation in ALT related to AAV gene therapy	Long et al (2021)^14^
Duchenne muscular dystrophy	Human (*n* = 4)	IV	Prednisolone (1 mg/kg)	Patients were on stable dose of corticosteroids for at least 12 weeks before study. Daily prednisone was started 1 day before AAV therapy with a 30-day taper post-AAV therapy	Not reported	Not reported	Mendell et al (2020)^20^
Hemophilia B	Human (*n* = 10)	IV	Prednisolone (60 mg/kg)	Patients with elevated ALT received a tapering dose of prednisolone	A transient increase in ALT occurred between week 7 and 10 in four of the six patients in the high-dose group but resolved after prednisolone treatment	Transient elevation in ALT related to AAV gene therapy	Nathwani et al (2014)^16^
Spinal muscular atrophy type 1	Human (*n* = 15)	IV	Prednisolone (1 mg/kg)	Fourteen patients received oral prednisone 1 day before AAV therapy then tapered for ∼30 days. One patient with elevated liver enzymes received additional prednisolone treatment	One patient did not receive prophylactic prednisolone and experienced elevated AST and ALT leading to a protocol amendment	Transient elevation in ALT/AST attenuated by prednisolone treatment before AAV infusion	Mendell et al (2017)^21^
Amyotrophic lateral sclerosis	Human (*n* = 2)	IT	Patient 1: methylprednisolone (1 g) Prednisone (60 mg/day)	Methylprednisolone was given the day of and 1 day after AAV therapy followed by prednisone tapered over a 4-week period	Meningoradiculitis	Death	Mueller (2020)^134^
			Patient 2: Rituximab (375 mg/m^2^) Methylprednisolone (125 mg) Prednisone (0.5 mg/kg) Rapamycin (6 mg)	Rituximab and methylprednisolone were given weekly for 3 weeks. Rapamycin was started the day of AAV therapy and prednisolone was started the day after AAV therapy; both were continued for 6 months	Blunted generation of nAbs, antiviral Abs, and T cell response to the viral capsid. Liver enzymes were not elevated, no sensory dysfunction, and no CSF pleocytosis	Not reported	
Pompe disease	Human (*n* = 9)	IM	Rituximab (1,125–1,500 mg/m^2^) Rapamycin (0.06–1 mg/m^2^) Methylprednisolone (10 mg/kg)	Rituximab, methylprednisolone, rapamycin, IVIG, and ERT were given before AAV therapy. Methylprednisolone was given the day of AAV therapy and continued for 3 days post-AAV therapy	Anticapsid and antitransgene responses occurred in all subjects *not* receiving concomitant immunomodulation	Not reported	Byrne (2014)^135^ Corti et al (2017)^19^
Lipoprotein lipase deficiency	Human (*n* = 14)	IM	Cyclosporine (3 mg/kg/day) MMF (2 g/day)	Cyclosporine and MMF were initiated at time of AAV therapy and continued for 12 weeks	Treatment-emergent increase in anti-AAV antibodies were *not* affected by immune suppression. A moderate nonpersistent T cell response was observed directed against the AAV capsid in 9 of 14 subjects	Immunosuppression did not impact biochemical/inflammatory markers	Gaudet (2013)^136^
N/A (GFP)	Nonhuman primate (*n* = 6)	ICV or IT	Rapamycin (2 mg/kg/day) Prednisolone 1–2 mg/kg/day) MMF (40 mg/kg/day)	Dosing details not provided	All subjects were positive for neutralizing factors and CD8^+^ T cell response in three of six subjects	Not reported	Bey (2020)^137^
Hunter syndrome (lysosomal enzyme deficiency)	Nonhuman primate (*n* = 12)	ICM	Rapamycin (0.75–2 mg/kg) MMF (25–100 mg/kg bid)	Rapamycin and MMF were given 14–21 days before AAV therapy. MMF was continued for 60 days. Rapamycin was continued for 90 days	Immunosuppression prevented pleocytosis but did not prevent neuronal degeneration	Immunosuppression-related adverse gastrointestinal effects and anemia. One subject had elevated transaminases	Hordeaux et al (2018)^94^
Duchenne muscular dystrophy	Nonhuman primate (*n* = 36)	Isolated limb perfusion	Prednisone (0.75 mg/kg/day) Tacrolimus (2 mg/kg/day) MMF (50 mg/kg/day)	Immunosuppression started 2 weeks before AAV therapy. Prednisone only and a prednisone/tacrolimus/MMF combination. Tacrolimus and MMF were continued for 12 weeks. Prednisone was continued for 24 weeks	No observable benefit of immunosuppression on transgene expression or AAV-binding antibodies	None reported	Chicoine (2014)^138^
Duchenne muscular dystrophy	Nonhuman primate (*n* = 25)	IM	Tacrolimus (0.06 mg/kg)	Immunosuppression started 3 days before AAV therapy and continued throughout the study (42 weeks)	Tacrolimus regulated the immune response and decreased IgM generation to the transgene product	No significant adverse events	Ishii (2020)^139^
Acute intermittent porphyria	Nonhuman primate (*n* = 3)	IV	Rituximab (20 mg/kg/dose) Tacrolimus (0.25 mg/kg/day) MMF (25 mg/kg) Methylprednisolone ATG (3 mg/kg)	Rituximab started 9 days before AAV therapy. ATG, tacrolimus, methylprednisolone, and MMF started 2 days before AAV therapy. Rituximab, tacrolimus, and MMF continued for 12 weeks	Immunosuppression regimen blunted humoral response and abolished T cell response to AAV. On withdrawal of immunosuppression, anticapsid nAb titers increased	MMF-dependent drug-mediated interference with liver transgene expression	Unzu (2012)^140^
Heart failure	Mini pig (*n* = 24)	Intracoronary	Rapamycin (2 mg/animal) Methylprednisolone (10 mg/kg for 30 days [from day 0 to 29]), followed by 5 mg/kg for an additional 76 days (from day 30 to 105) MMF (250 mg/animal)	MMF and rapamycin started 14 days before AAV therapy and continued to day 105. Methylprednisolone started the day of AAV therapy and continued to day 105	Immunosuppression regimen did not prevent the development of neutralizing antibodies	None reported	Greenberg (2016)^141^

ALT, alanine aminotransferase; AST, aspartate aminotransferase; ATG, antithymocyte gamma-globulin; CSF, cerebrospinal fluid; ERT, enzyme replacement therapy; GFP, green fluorescent protein; ICM, intra-cisterna magna; ICV, intracerebroventricular; IgM, immunoglobulin G; IM, intramuscular; IT, intrathecal; IV, intravenous; IVIG, intravenous immunoglobulin; MMF, mycophenolate mofetil; N/A, not applicable; nAb, neutralizing antibody.

Clinical and preclinical studies indicating the use of immunosuppressants are summarized in Table 2. Data from the clinical studies suggest that prophylactic administration of immunosuppressants may attenuate some adverse immunological responses to AAV gene therapy. It is important to note that several different endpoints were used (*e.g.,* T cell response [ELISpot], liver enzyme [transaminase] levels, and capsid- or transgene-specific responses [enzyme-linked immunosorbent assay]), and given the absence of a control group, these results should be interpreted with caution. Preclinical studies must also be interpreted with caution as the immune responses in animal models are not always predictive of human outcomes. Several clinical trials using immunosuppression regimens aimed at treating CNS disorders are currently in progress (NCT03952637, NCT04669535, NCT03381729, NCT03199469, NCT03533673, NCT04833907, NCT04411654, NCT04408625).

## CONCLUSIONS

AAVs are used as vectors for developing gene therapies that can be directly delivered to CNS to treat CNS disorders. The innate and adaptive immune responses may pose a barrier to safe and effective AAV gene therapy. Preexisting nAbs to AAV capsid proteins can prevent target cell transduction, thereby limiting the efficacy of gene therapy, and prevent redosing. The cytotoxic aspect of T cell activation can cause transduced cell damage resulting in hepatotoxicity, neurotoxicity, and loss of transgene expression.

Available evidence, mostly from small animal and NHP studies, suggests that pleocytosis, dorsal root ganglionitis, dorsal root ganglia degeneration, TMA, and liver toxicity are potential concerns for the safety of CNS-administered AAV gene therapies. In addition, systemic administration of some of the AAV serotypes that cross the BBB could potentially lead to neuronal toxicity. Consequently, multiple studies in NHPs and humans have used pharmacological immunomodulation in attempts to limit toxicity and increase efficacy. Regrettably, most studies, especially in humans, have significant methodological challenges, most notably a lack of control groups and a lack of data on the total antibody response (*i.e.,* titer). For rational selection of immunomodulatory therapy, further mechanistic studies are required to understand the relative contributions of the innate immune system (most notably DAMPs and PAMPs) in model systems with similar immune systems to humans, in addition to broader data collection during current and proposed human trials.

Current approaches to limit the potential for toxicity, immunogenicity, and reduced transduction efficiency of AAV gene therapy include the exclusion of subjects with preexisting anticapsid nAbs (>1:5) or with profound preexisting immune dysregulation. Other tactics include vector design and manufacture considerations such as choice of AAV serotype, promoter design, reduction of CpG islands, elimination of impurities, and empty capsids. Immunomodulatory strategies, such as broad immunosuppression with corticosteroid administration before and after AAV dosing, interference with cytokine/inflammatory signaling (*e.g.,* rapamycin), T cell suppression (*e.g.,* MMF, calcineurin inhibitors), B cell suppression (*e.g.,* MMF, rituximab), complement suppression (*e.g.,* eculizumab), and drugs that alter TLR9 signaling (*e.g.,* hydroxychloroquine) should be carefully considered because they can also be associated with adverse effects. Systematic clinical monitoring and reporting of immunological events will help guide the development of immunosuppressant regimens for future trials.

Based on published preclinical and clinical data, it is likely that immunosuppressive therapy is needed to maximize the safety and efficacy of gene therapy. Although several options are available, appropriate pharmacological intervention should carefully balance effective dampening of the innate and adaptive immune responses to gene therapy while attempting to minimize the adverse effects associated with immunosuppression. The impact of compromising host–defense mechanisms with immunosuppression adds to the importance of developing a well-designed monitoring plan. Key considerations during clinical trials include monitoring of adverse events and having appropriate protocols in place to treat breakthrough immunological events. Understanding the mechanisms that drive these events will guide the proper use of immunosuppressive therapies and help to inform future studies and clinical trials.

Although the topic of the use of immunosuppression is an evolving discussion with many different perspectives, we recommend that investigators consider the practical questions given in Table 3 before starting patients on a clinical trial for AAV gene therapy. Answers to these questions will vary depending on many factors including, but not limited to, route of administration, CRIM status, preexisting nAbs, patient age, disease progression, and comorbidities. Thorough consideration of these factors in the context of immunosuppression will help ensure the safety of the patients and the efficacy of AAV gene therapy. It is important to remember that gene therapy is a rapidly evolving field, and current immunosuppression strategies will likely change as more data become available.

**Table 3.** List of practical questions to consider before starting patients on a clinical trial for gene therapy

1	How much immunosuppression is needed? Specifically, what serum target levels of the immunosuppressant medication(s) are required for adequate immunosuppression?
2	Which specific immunosuppressant medication(s) are needed? Consider the class (*e.g.,* B cell ablator, T cell modifier, corticosteroid, anticomplement factor) and the drug itself
3	When should each immunosuppressant medication begin in relation to the initiation of gene therapy?
4	What immunizations are needed before commencing gene therapy, and how will additional immunizations be managed during the trial?
5	Should the patient be prescribed concomitant prophylactic antimicrobials/antifungals/antivirals?
6	What monitoring should occur while the patient is on immunosuppressive medication? Monitoring is likely to be the most intense around the initiation of gene therapy and will diminish over time
7	How long should the patient remain on immunosuppressant medication(s)? The duration may be different for each immunosuppressant
8	How will breakthrough immunological events be monitored for and how frequently?
9	How does the immunosuppression regimen need to be altered in the case of a breakthrough immunological event? Consider the degree of the event and the rapidity of progression. What other management might be required, and what tests would be needed?
10	How will a breakthrough infective event be monitored for and treated, and how (if at all) will the immunosuppressive medication be modified under this circumstance?
11	How will the immunosuppressive regimen be modified if there is an adverse event to one of the immunosuppressive medications?
12	How does the immunosuppression regimen need to be altered in the case of an immunological event caused by the gene therapy? Consider the degree of the event and the rapidity of deterioration
13	How will the immunosuppressant medication(s) be tapered and over what time frame? What is the monitoring plan during the tapering period? Consider criteria for interrupting the taper or reintroducing immunosuppressive agents if necessary
14	Does the Data Safety Monitoring Board have adequate and appropriate expertise? Is there access to appropriate and experienced advisors across a range of specialties (*e.g.,* hepatology, hematology, cardiology)?
